# The genome sequence of the small pearl-bordered fritillary butterfly, *Boloria selene *(Schiffermüller, 1775)

**DOI:** 10.12688/wellcomeopenres.17734.1

**Published:** 2022-03-04

**Authors:** Konrad Lohse, Derek Setter, Darwin Tree of Life Barcoding collective, Wellcome Sanger Institute Tree of Life programme, Wellcome Sanger Institute Scientific Operations: DNA Pipelines collective, Tree of Life Core Informatics collective, Darwin Tree of Life Consortium

**Affiliations:** 1 Institute of Evolutionary Biology, University of Edinburgh, Edinburgh, UK

**Keywords:** Boloria selene, small pearl-bordered fritillary, silver meadow fritillary, genome sequence, chromosomal, Lepidoptera

## Abstract

We present a genome assembly from an individual female *Boloria selene *(the small pearl-bordered fritillary, also known as the silver meadow fritillary; Arthropoda; Insecta; Lepidoptera; Nymphalidae). The genome sequence is 400 megabases in span. The complete assembly is scaffolded into 31 chromosomal pseudomolecules, with the W and Z sex chromosome assembled.

## Species taxonomy

Eukaryota; Metazoa; Ecdysozoa; Arthropoda; Hexapoda; Insecta; Pterygota; Neoptera; Endopterygota; Lepidoptera; Glossata; Ditrysia; Papilionoidea; Nymphalidae; Heliconiinae; Argynnini; Boloria; *Boloria selene* (Schiffermüller, 1775) (NCBI:txid191398).

## Background

The small pearl-bordered fritillary ( *Boloria selene*) is a widespread butterfly of boreal habitats in Northern Europe, North America, and Asia ( Filz *et al.*, 2012; Roy *et al.*, 2015). Nearctic and Palaearctic populations have been estimated to have diverged during the early Pleistocene, approximately 2.5 Mya ( Maresova *et al.*, 2019). Within the UK, *B. selene* is endemic to mainland Scotland, Wales, and the West coast of England. The species is known as *enw gwyddonol* in Welsh and *an neamhnaideach beag* in Scottish Gaelic. Larvae feed exclusively on violets ( Swengel, 1997), while adults use a wide range of floral resources including buttercups and bird’s-foot-trefoil ( Tudor *et al.*, 2004) and may migrate short distances for reproduction ( Dapporto & Dennis, 2013; Kuussaari *et al.*, 2014). *B. selene* is a species that inhabits damp grassland clearings within deciduous woodland where the larval food source is abundant ( Filz *et al.*, 2012). Although the species is classed as Least Concern in the IUCN Red List ( van Swaay *et al.*, 2010), habitat loss and fragmentation due to agriculture in combination with diet specificity and limited dispersal have led to significant population declines over the last half-century ( Filz *et al.*, 2012). In the UK, *B. selene* is classified as a priority species under the Biodiversity Action Plan ( Fox *et al.*, 2015; Stewart *et al.*, 2004). *B. selene* has 30 chromosome pairs ( Federley, 1938; Lorković, 1941).

## Genome sequence report

The genome was sequenced from a single female *B. selene* collected from Carrifran Wildwood, Scotland (latitude 55.4001, longitude -3.3352). A total of 60-fold coverage in Pacific Biosciences single-molecule circular consensus (HiFi) long reads and 77-fold coverage in 10X Genomics read clouds were generated. Primary assembly contigs were scaffolded with chromosome conformation Hi-C data. Manual assembly curation corrected 8 missing/misjoins, reducing the scaffold number by 16.22% and increasing the scaffold N50 by 0.16%.

The final assembly has a total length of 400 Mb in 31 sequence scaffolds with a scaffold N50 of 13.6 Mb ( Table 1). The complete assembly sequence was assigned to 31 chromosomal-level scaffolds, representing 29 autosomes (numbered by sequence length), and the W and Z sex chromosome ( Figure 2– Figure 5; Table 2). The assembly has a BUSCO v5.1.2 ( Manni *et al.*, 2021) completeness of 98.6% (single 98.3%, duplicated 0.3%) using the lepidoptera_odb10 reference set (n=5286). While not fully phased, the assembly deposited is of one haplotype. Contigs corresponding to the second haplotype have also been deposited.

**Table 1.** Genome data for *Boloria selene*, ilBolSele5.2.

*Project accession data*	
Assembly identifier	ilBolSele5.2
Species	*Boloria selene*
Specimen	ilBolSele5 (genome assembly, Hi-C), ilBolSele2 (additional 10X reads)
NCBI taxonomy ID	NCBI:txid191398
BioProject	PRJEB43033
BioSample ID	SAMEA7523131
Isolate information	Female, whole organism (ilBolSele5); male, whole organism (ilBolSele2)
*Raw data accessions*	
PacificBiosciences SEQUEL II	ERR6412358
10X Genomics Illumina	ERR6054466-ERR6054469 (ilBolSele5); ERR6054462- ERR6054465 (ilBolSele2)
Hi-C Illumina	ERR6054470
*Genome assembly*	
Assembly accession	GCA_905231865.2
*Accession of alternate haplotype*	GCA_905231875.1
Span (Mb)	400
Number of contigs	41
Contig N50 length (Mb)	13.4
Number of scaffolds	31
Scaffold N50 length (Mb)	13.6
Longest scaffold (Mb)	16.1
BUSCO * genome score	C:98.6%[S:98.3%,D:0.3%],F: 0.2%,M:1.2%,n:5286

*BUSCO scores based on the lepidoptera_odb10 BUSCO set using v5.1.2. C= complete [S= single copy, D=duplicated], F=fragmented, M=missing, n=number of orthologues in comparison. A full set of BUSCO scores is available at https://blobtoolkit.genomehubs.org/view/ilBolSele5.2/dataset/ilBolSele5_2/busco.

**Table 2.** Chromosomal pseudomolecules in the genome assembly of *Boloria selene*, ilBolSele5.2.

INSDC accession	Chromosome	Size (Mb)	GC%
HG993132.1	1	16.08	32.5
HG993133.1	2	16.07	32.8
HG993134.1	3	15.71	32.8
HG993135.1	4	15.18	32.7
HG993137.1	5	15.16	32.6
HG993138.1	6	15.13	32.4
HG993139.1	7	14.44	32.4
HG993140.1	8	14.43	32.3
HG993141.1	9	14.36	32.5
HG993142.1	10	13.68	32.2
HG993143.1	11	13.62	32.1
HG993144.1	12	13.60	32.6
HG993145.1	13	13.40	32.4
HG993146.1	14	13.16	32.2
HG993147.1	15	12.92	32.5
HG993148.1	16	12.72	32.5
HG993149.1	17	12.53	32.6
HG993150.1	18	12.34	32.8
HG993151.1	19	11.81	33.1
HG993152.1	20	11.69	32.5
HG993153.1	21	11.14	32.7
HG993154.1	22	10.33	33.4
HG993155.1	23	10.05	32.7
HG993156.1	24	9.14	32.8
HG993157.1	25	9.10	32.5
HG993158.1	26	8.95	34.1
HG993159.1	27	8.17	35.4
HG993160.1	28	6.92	35.0
HG993161.1	29	6.91	34.2
HG993136.1	W	15.17	36.7
HG993131.1	Z	25.99	32.0
HG998571.1	MT	0.02	19.0

## Methods

### Sample acquisition, DNA extraction and sequencing

**Figure 1.**
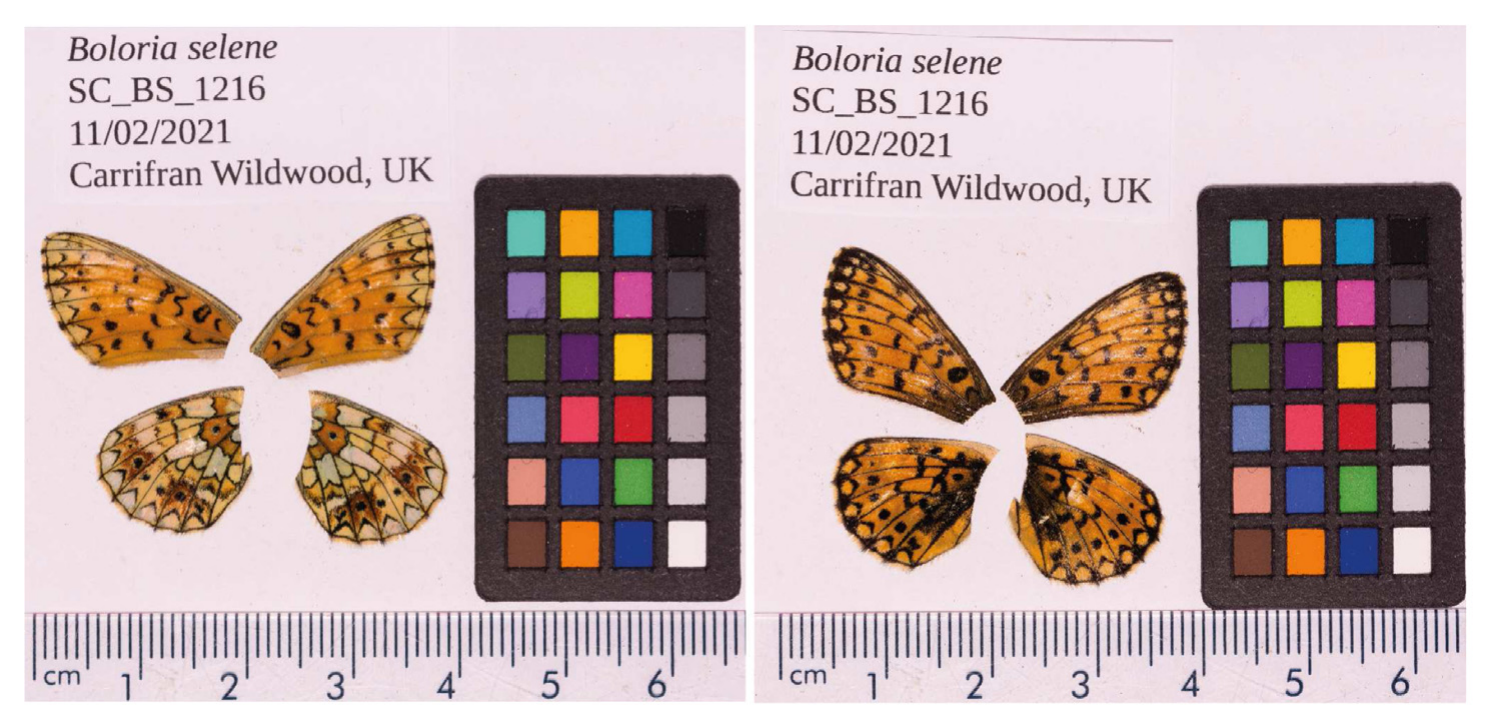
Fore and hind wings of the *Boloria selene* specimen from which the genome was sequenced. Dorsal (left) and ventral (right) surface view of wings from specimen UK_BS_1216 (ilBolSele5) from Carrifran Wildwood, Scotland, used to generate 10X, HiFi and Hi-C data.

A female *B. selene* specimen (ilBolSele5) was collected from Carrifran Wildwood, Scotland (latitude 55.4001, longitude -3.3352) using a net by Konrad Lohse, who also identified the sample. Another male specimen (ilBolSele2; Figure 1) was also collected from the same location by the same individual. Specimens were snap-frozen at -80°C.

**Figure 2.**
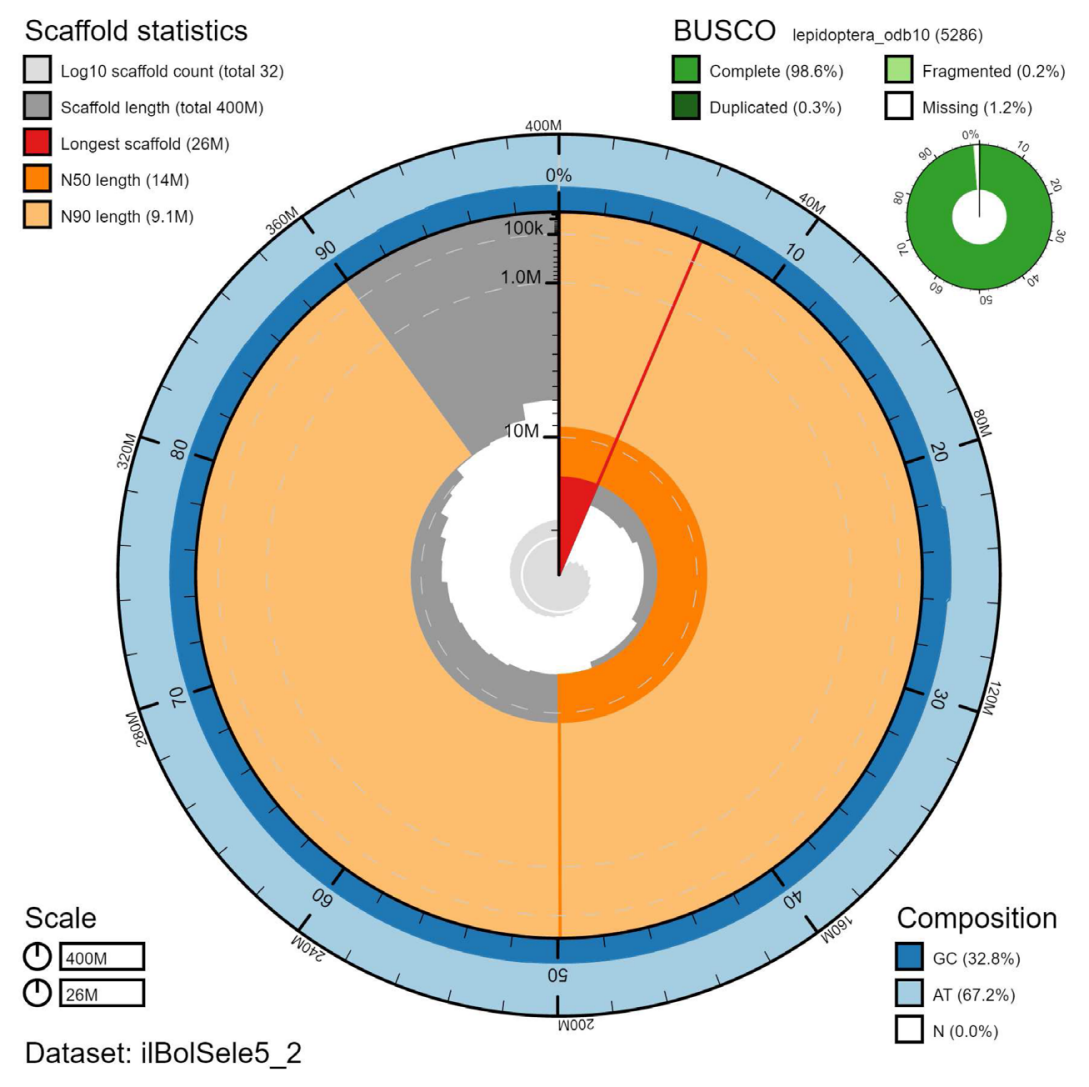
Genome assembly of *Boloria selene*, ilBolSele5.2: metrics. The BlobToolKit Snailplot shows N50 metrics and BUSCO gene completeness. The main plot is divided into 1,000 size-ordered bins around the circumference with each bin representing 0.1% of the 399,917,640 bp assembly. The distribution of chromosome lengths is shown in dark grey with the plot radius scaled to the longest chromosome present in the assembly (25,989,679 bp, shown in red). Orange and pale-orange arcs show the N50 and N90 chromosome lengths (13,620,786 and 9,102,699 bp), respectively. The pale grey spiral shows the cumulative chromosome count on a log scale with white scale lines showing successive orders of magnitude. The blue and pale-blue area around the outside of the plot shows the distribution of GC, AT and N percentages in the same bins as the inner plot. A summary of complete, fragmented, duplicated and missing BUSCO genes in the lepidoptera_odb10 set is shown in the top right. An interactive version of this figure is available at https://blobtoolkit.genomehubs.org/view/ilBolSele5.2/dataset/ilBolSele5_2/snail.

**Figure 3.**
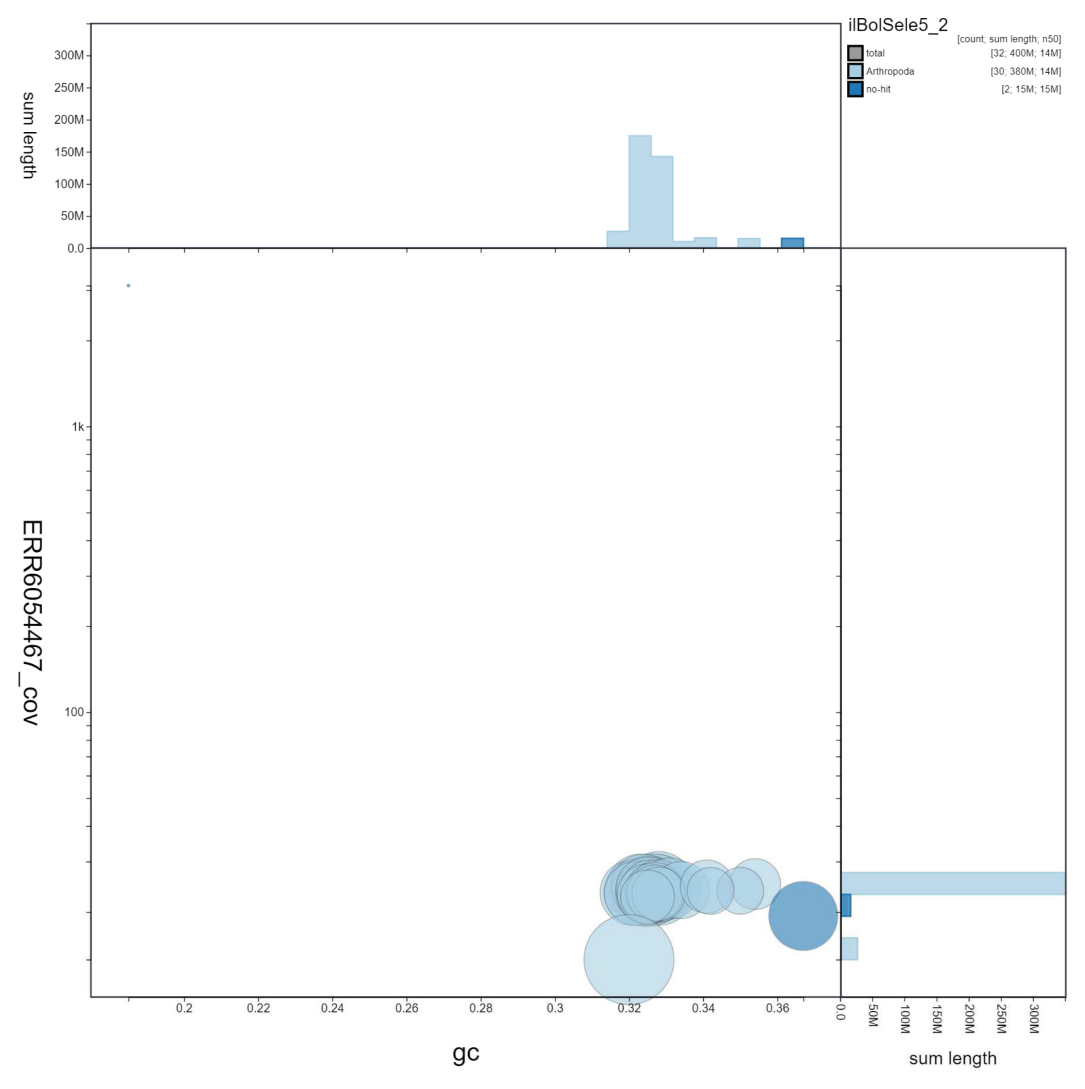
Genome assembly of *Boloria selene*, ilBolSele5.2: GC coverage. BlobToolKit GC-coverage plot. Scaffolds are coloured by phylum. Circles are sized in proportion to scaffold length. Histograms show the distribution of scaffold length sum along each axis. An interactive version of this figure is available at https://blobtoolkit.genomehubs.org/view/ilBolSele5.2/dataset/ilBolSele5_2/blob

**Figure 4.**
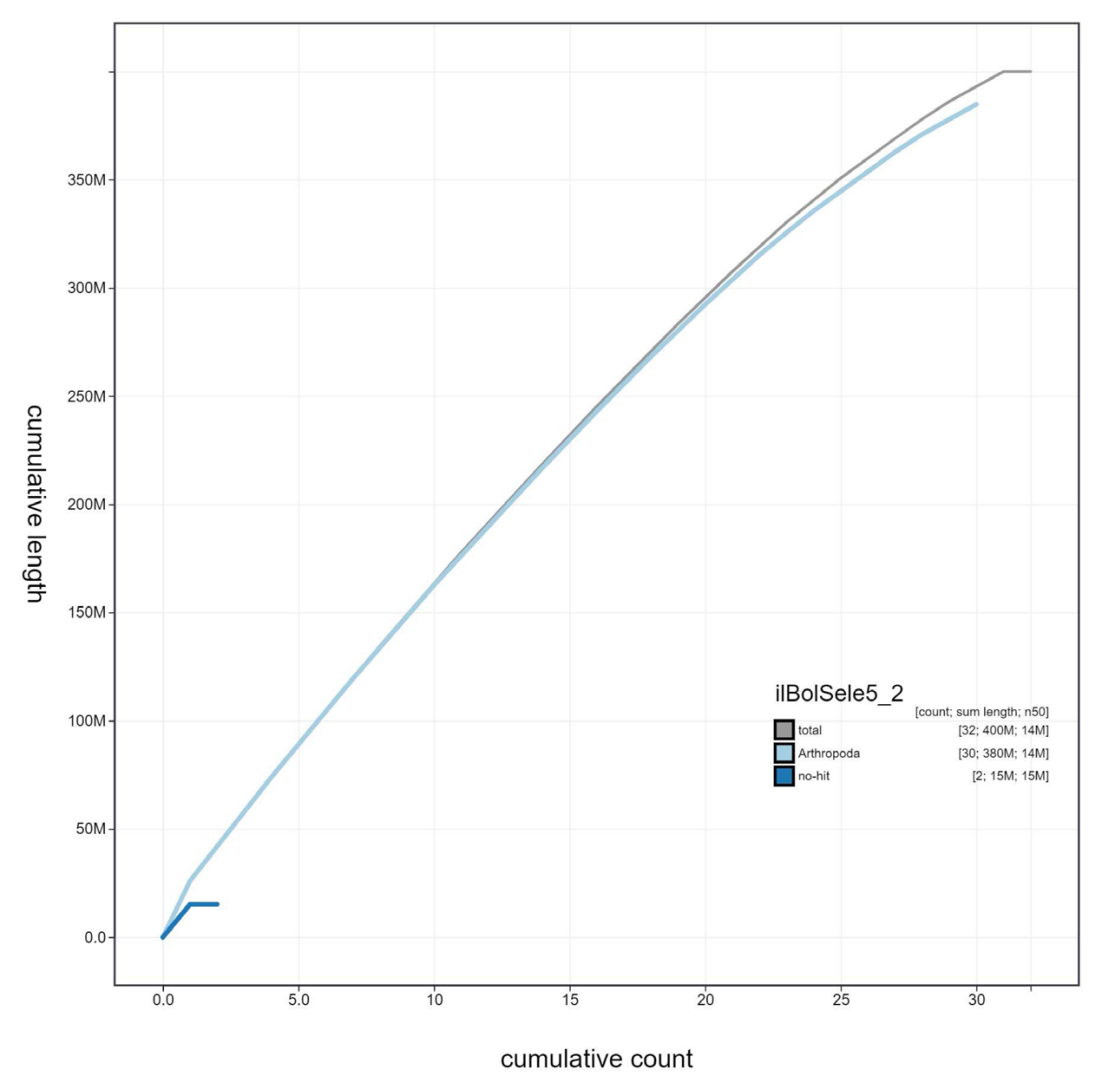
Genome assembly of *Boloria selene*, ilBolSele5.2: cumulative sequence. BlobToolKit cumulative sequence plot. The grey line shows cumulative length for all scaffolds. Coloured lines show cumulative lengths of scaffolds assigned to each phylum using the buscogenes taxrule. An interactive version of this figure is available at https://blobtoolkit.genomehubs.org/view/ilBolSele5.2/dataset/ilBolSele5_2/cumulative.

**Figure 5.**
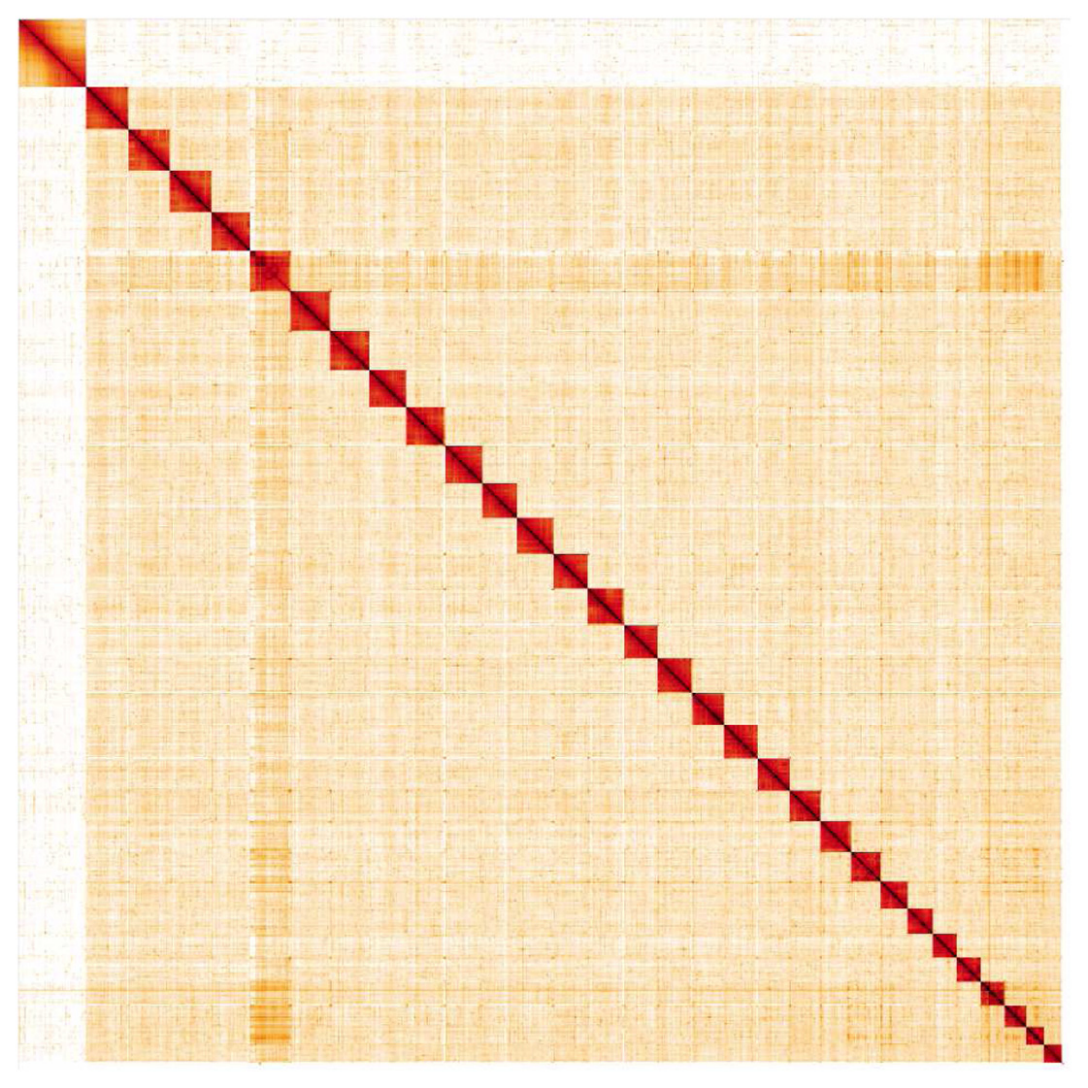
Genome assembly of *Boloria selene*, ilBolSele5.2: Hi-C contact map. Hi-C contact map of the ilBolSele5.2 assembly, visualised in HiGlass. Chromosomes are shown in size order from left to right and top to bottom.

DNA was extracted from the whole organism of ilBolSele5 and ilBolSele2 at the Wellcome Sanger Institute (WSI) Scientific Operations core from the whole organism using the Qiagen MagAttract HMW DNA kit, according to the manufacturer’s instructions. Pacific Biosciences HiFi circular consensus (for ilBolSele5) and 10X Genomics read cloud (ilBolSele5 and ilBolSele2) DNA sequencing libraries were constructed according to the manufacturers’ instructions. DNA and RNA sequencing was performed by the Scientific Operations core at the WSI on Pacific Biosciences SEQUEL II (HiFi) and Illumina HiSeq X (10X) instruments. Hi-C data were also generated from remaining whole organism tissue of ilBolSele5 using the Arima v2 Hi-C kit and sequenced on an Illumina NovaSeq 6000 instrument.

### Genome assembly

Assembly was carried out with Hifiasm ( Cheng *et al.*, 2021) haplotypic duplication was identified and removed with purge_dups ( Guan *et al.*, 2020). One round of polishing was performed by aligning 10X Genomics read data to the assembly with longranger align, calling variants with freebayes ( Garrison & Marth, 2012). The assembly was then scaffolded with Hi-C data ( Rao *et al.*, 2014) using SALSA2 ( Ghurye *et al.*, 2019). The assembly was checked for contamination and corrected using the gEVAL system ( Chow *et al.*, 2016) as described previously ( Howe *et al.*, 2021). Manual curation ( Howe *et al.*, 2021) was performed using gEVAL, HiGlass ( Kerpedjiev *et al.*, 2018) and Pretext. The mitochondrial genome was assembled using MitoHiFi ( Uliano-Silva *et al.*, 2021), which performed annotation using MitoFinder ( Allio *et al.*, 2020). The genome was analysed and BUSCO scores generated within the BlobToolKit environment ( Challis *et al.*, 2020). Table 3 contains a list of all software tool versions used, where appropriate.

**Table 3.** Software tools used.

Software tool	Version	Source
Hifiasm	0.12	Cheng *et al.*, 2021
purge_dups	1.2.3	Guan *et al.*, 2020
SALSA2	2.2	Ghurye *et al.*, 2019
longranger align	2.2.2	https://support.10xgenomics.com/ genome-exome/software/pipelines/ latest/advanced/other-pipelines
freebayes	1.3.1-17-gaa2ace8	Garrison & Marth, 2012
MitoHiFi	1	Uliano-Silva *et al.*, 2021
gEVAL	N/A	Chow *et al.*, 2016
HiGlass	1.11.6	Kerpedjiev *et al.*, 2018
PretextView	0.1.x	https://github.com/wtsi-hpag/PretextView
BlobToolKit	2.6.4	Challis *et al.*, 2020

## Data availability

European Nucleotide Archive: Boloria selene (silver meadow fritillary). Accession number PRJEB43033; https://identifiers.org/ena.embl/PRJEB43033.

The genome sequence is released openly for reuse. The *B. selene* genome sequencing initiative is part of the Darwin Tree of Life (DToL) project. The genome will be annotated and presented through the Ensembl pipeline at the European Bioinformatics Institute. All raw sequence data and the assembly have been deposited in INSDC databases. Raw data and assembly accession identifiers are reported in Table 1.

## Author information

Members of the Darwin Tree of Life Barcoding collective are listed here: https://doi.org/10.5281/zenodo.5744972.

Members of the Wellcome Sanger Institute Tree of Life programme are listed here: https://doi.org/10.5281/zenodo.6125027.

Members of Wellcome Sanger Institute Scientific Operations: DNA Pipelines collective are listed here: https://doi.org/10.5281/zenodo.5746904.

Members of the Tree of Life Core Informatics collective are listed here: https://doi.org/10.5281/zenodo.6125046.

Members of the Darwin Tree of Life Consortium are listed here: https://doi.org/10.5281/zenodo.5638618.
