# Supplementary material for: The genome sequence of the small pearl-bordered fritillary butterfly, Boloria selene (Schiffermüller, 1775)
Source: Wellcome Open Res. 2022 Mar 4;7:76. [Version 1] doi: 10.12688/wellcomeopenres.17734.1 (PMC9713055; doi:10.12688/wellcomeopenres.17734.1)
Supplement: Peer review history

# Peer review: Reviewer response for version 1

**DOI:** 10.21956/wellcomeopenres.19625.r53306

**Published:** 2022-11-30

**Authors:** Vladimir A Lukhtanov

**Affiliations:** 1 Department of Karyosystematics, Zoological Institute of Russian Academy of Sciences, St. Petersburg, Russian Federation

The authors present a chromosome-scale genome assembly for the small pearl-bordered fritillary, *Boloria selene*. The note provides brief information about the species, its genome, and the methods that were used to sequence and assemble the genome. This high-quality assembly will serve as a tool for a variety of studies as *Boloria selene* is a model for ecological and evolutionary research. It is also important to note that it is an extremely widespread species across three continents: Asia, North America, and Europe. Therefore, as a result of the presented study, scientists from many countries have received free access to the necessary and useful genetic resource. The presented note will serve as a starting point for all these future studies.

 I have the following minor corrections and suggestions:

**Section “Species taxonomy”**

 “Boloria” should be written in italics.

 Written: *Boloria selene* (Schiffermüller, 1775). Should be: *Boloria selene* ([Denis & Schiffermüller], 1775) (for example, see Wiemers et al. 2018) ^1^.

**Background**

 “Larvae feed exclusively on violets” please correct to “Larvae feed exclusively on violets *(* *Viola* *)* *” (*scientific name is required).

 “while adults use a wide range of floral resources including buttercups and bird’s-foot-trefoil” please correct to “while adults use a wide range of floral resources including buttercups ( *Ranunculus*) and bird’s-foot-trefoil ( *Lotus corniculatus*)” *(*scientific names are required).

**Genome sequence report**

 Not a word is said about the mitochondrial genome. It is completely forgotten.

 It would be valuable to point out that Z is the largest chromosome in the set.

**Methods**

 In the caption to Figure 1, “Dorsal (left) and ventral (right) surface” please replace by  “Ventral (left) and dorsal (right) surface”.

**Data availability**

 Boloria selene should be in italics.

**References**

 Federley: “Finn-ländischer” please correct to  “Finnländischer”

Are sufficient details of methods and materials provided to allow replication by others?

Yes

Is the rationale for creating the dataset(s) clearly described?

Yes

Are the datasets clearly presented in a useable and accessible format?

Yes

Are the protocols appropriate and is the work technically sound?

Yes

Reviewer Expertise:

Cytogenetics, phylogenetics, Lepidoptera taxonomy and systematics, evolutionary biology

I confirm that I have read this submission and believe that I have an appropriate level of expertise to confirm that it is of an acceptable scientific standard.

## References

[1] : An updated checklist of the European Butterflies (Lepidoptera, Papilionoidea). *Zookeys* .2018; 10.3897/zookeys.811.28712 9-45 10.3897/zookeys.811.28712 30627036PMC6323101

# Peer review: Reviewer response for version 1

**DOI:** 10.21956/wellcomeopenres.19625.r49247

**Published:** 2022-04-12

**Authors:** Zdenek Faltynek Fric

**Affiliations:** 1 Institute of Entomology, Biology Centre CAS, České Budějovice, Czech Republic

The authors publish the complete genome of *Boloria selene*. They describe the origin of the sample, which is very important due to the large distribution of the species, and the authors also sufficiently but in detail explain all procedures.

 It seems only that in the "Genome assembly" chapter, the first sentence, a part of the text was dropped and thus two sentences are merged together.

 Otherwise, I see the initiative as very important and it is very useful if the reference genome of this butterfly species is available.

Are sufficient details of methods and materials provided to allow replication by others?

Yes

Is the rationale for creating the dataset(s) clearly described?

Yes

Are the datasets clearly presented in a useable and accessible format?

Yes

Are the protocols appropriate and is the work technically sound?

Yes

Reviewer Expertise:

Lépidoptera phylogeny and phylogeography, butterfly ecology

I confirm that I have read this submission and believe that I have an appropriate level of expertise to confirm that it is of an acceptable scientific standard.
